# Supplementary material for: A Likelihood Approach for Real-Time Calibration of Stochastic Compartmental Epidemic Models
Source: PLoS Comput Biol. 2017 Jan 17;13(1):e1005257. doi: 10.1371/journal.pcbi.1005257 (PMC5240920; doi:10.1371/journal.pcbi.1005257)
Supplement: S1 File — (TAR.GZ) [file pcbi.1005257.s014.tar.gz › HSPH_Online-SI-Revision/Additions/PF09/n100-extreme-SEITR/n100-extreme-SEITR_PF2_table-8.pdf]

1	newly 3 weeks spec	2187.	579.38	{462., 724.}	73.508	0.582843	newly 3 weeks cumul	3760.	1359.75	{1120., 1680.}	63.8364	0.446715	newly 5 weeks spec	7106.	972.26	{770., 1232.}	86.3178	0.870526	newly 5 weeks cumul	14965.	3085.68	{2514., 3852.}	79.3807	0.691362	newly 59 weeks cumul	91886.	73208.9	{71064., 75308.}	20.3264	0.0988371
2	newly 3 weeks spec	265.	342.27	{76., 724.}	81.	0.318733	newly 3 weeks cumul	343.	680.76	{189., 1365.}	122.776	0.319587	newly 5 weeks spec	3034.	954.42	{136., 2367.}	75.408	0.798005	newly 5 weeks cumul	4334.	2211.19	{447., 5114.}	66.3459	0.544857	newly 66 weeks cumul	99017.	72281.1	{45585., 92195.}	27.0014	0.150943
3	newly 3 weeks spec	257.	538.01	{14., 1184.}	188.214	0.665213	newly 3 weeks cumul	379.	891.92	{32., 1949.}	191.573	0.587995	newly 5 weeks spec	2684.	1888.42	{20., 5827.}	109.169	1.10963	newly 5 weeks cumul	3894.	3897.61	{66., 10550.}	120.163	0.890821	newly 68 weeks cumul	98993.	61596.3	{23478., 89885.}	37.7772	0.480871
4	newly 3 weeks spec	698.	343.52	{88., 676.}	61.8052	0.481121	newly 3 weeks cumul	1030.	692.43	{201., 1336.}	52.0204	0.340026	newly 5 weeks spec	3933.	919.07	{163., 2391.}	79.1561	0.870849	newly 5 weeks cumul	6733.	2176.05	{479., 4872.}	71.6921	0.672618	newly 66 weeks cumul	96862.	70105.4	{44724., 92045.}	27.6234	0.156959
5	newly 3 weeks spec	187.	529.33	{10., 1184.}	247.813	0.660588	newly 3 weeks cumul	237.	871.5	{29., 1848.}	306.962	0.639727	newly 5 weeks spec	3231.	1890.41	{15., 5827.}	102.244	1.20649	newly 5 weeks cumul	4188.	3875.28	{48., 10550.}	116.839	0.957176	newly 68 weeks cumul	98880.	61048.3	{15133., 89885.}	38.2602	0.539664
6	newly 3 weeks spec	323.	458.64	{65., 1001.}	103.975	0.37424	newly 3 weeks cumul	460.	832.26	{153., 1863.}	119.865	0.33093	newly 5 weeks spec	3690.	1472.97	{101., 4181.}	82.4285	0.868414	newly 5 weeks cumul	5281.	3167.21	{355., 7437.}	81.1485	0.635376	newly 67 weeks cumul	95041.	71901.3	{47376., 92761.}	24.347	0.135701
7	newly 3 weeks spec	2336.	653.07	{531., 797.}	72.0432	0.558807	newly 3 weeks cumul	4235.	1536.2	{1264., 1867.}	63.7261	0.444714	newly 5 weeks spec	6848.	1097.19	{873., 1360.}	83.9779	0.800994	newly 5 weeks cumul	15415.	3483.09	{2866., 4293.}	77.4045	0.650846	newly 59 weeks cumul	97367.	73667.3	{70794., 76091.}	24.3406	0.121339
8	newly 3 weeks spec	476.	389.59	{83., 802.}	66.7584	0.391267	newly 3 weeks cumul	697.	756.22	{208., 1433.}	64.4706	0.285622	newly 5 weeks spec	4729.	1091.66	{118., 2703.}	81.2599	0.940891	newly 5 weeks cumul	7052.	2508.83	{440., 5761.}	72.83	0.688794	newly 66 weeks cumul	99432.	71491.1	{43976., 92221.}	28.1005	0.158854
9	newly 3 weeks spec	88.	529.33	{10., 1184.}	537.875	0.702874	newly 3 weeks cumul	112.	871.5	{29., 1848.}	705.446	0.808725	newly 5 weeks spec	2227.	1890.41	{15., 5827.}	119.352	1.10328	newly 5 weeks cumul	2748.	3875.28	{48., 10550.}	151.156	0.86368	newly 68 weeks cumul	99354.	61048.3	{15133., 89885.}	38.5548	0.541741
10	newly 3 weeks spec	982.	400.68	{140., 809.}	60.279	0.483798	newly 3 weeks cumul	1566.	849.1	{338., 1583.}	49.8966	0.351252	newly 5 weeks spec	4646.	897.21	{204., 2227.}	80.6885	0.866501	newly 5 weeks cumul	8317.	2348.29	{700., 5146.}	71.7652	0.658443	newly 64 weeks cumul	99174.	72613.8	{58461., 89957.}	26.7814	0.141474
11	newly 3 weeks spec	578.	334.82	{172., 540.}	43.9585	0.286732	newly 3 weeks cumul	848.	748.64	{443., 1153.}	31.5755	0.158726	newly 5 weeks spec	4035.	635.18	{305., 1161.}	84.2582	0.865707	newly 5 weeks cumul	6425.	1839.81	{961., 3109.}	71.3648	0.588642	newly 63 weeks cumul	99600.	73913.1	{66730., 82608.}	25.7901	0.131023
12	newly 3 weeks spec	936.	316.82	{118., 587.}	66.1517	0.543084	newly 3 weeks cumul	1372.	689.72	{281., 1223.}	51.9111	0.363135	newly 5 weeks spec	5911.	651.95	{188., 1339.}	88.9706	1.07507	newly 5 weeks cumul	9781.	1795.31	{609., 3613.}	81.6449	0.81955	newly 64 weeks cumul	98924.	71412.	{58998., 80475.}	27.8112	0.145466
13	newly 3 weeks spec	632.	420.	{125., 985.}	55.8892	0.363502	newly 3 weeks cumul	864.	842.66	{306., 1803.}	48.7454	0.230523	newly 5 weeks spec	4789.	1095.03	{239., 3277.}	77.152	0.833142	newly 5 weeks cumul	7468.	2622.88	{768., 6775.}	66.4738	0.598952	newly 65 weeks cumul	99507.	75499.	{55996., 94150.}	24.127	0.127837
14	newly 3 weeks spec	1910.	765.14	{632., 912.}	59.9403	0.401299	newly 3 weeks cumul	3449.	1800.44	{1500., 2095.}	47.7982	0.285443	newly 5 weeks spec	5421.	1248.91	{993., 1483.}	76.9616	0.642018	newly 5 weeks cumul	12064.	4028.19	{3318., 4701.}	66.6098	0.479951	newly 58 weeks cumul	96176.	73266.4	{71524., 74994.}	23.8205	0.118305
15	newly 3 weeks spec	1491.	549.13	{446., 672.}	63.1704	0.439407	newly 3 weeks cumul	2425.	1302.82	{1064., 1586.}	46.2755	0.274398	newly 5 weeks spec	6031.	909.52	{709., 1105.}	84.9193	0.82798	newly 5 weeks cumul	11560.	2927.98	{2409., 3560.}	74.6715	0.601617	newly 59 weeks cumul	97278.	72470.	{69914., 74721.}	25.5021	0.127977
16	newly 3 weeks spec	1486.	476.88	{268., 773.}	67.9085	0.5309	newly 3 weeks cumul	2628.	1075.59	{659., 1693.}	59.0719	0.419139	newly 5 weeks spec	4920.	878.43	{438., 1572.}	82.1457	0.800091	newly 5 weeks cumul	10361.	2601.37	{1438., 4410.}	74.8927	0.640592	newly 62 weeks cumul	92728.	73737.1	{65792., 82485.}	20.4802	0.100843
17	newly 3 weeks spec	187.	529.33	{10., 1184.}	247.813	0.660588	newly 3 weeks cumul	247.	871.5	{29., 1848.}	293.053	0.633156	newly 5 weeks spec	2188.	1890.41	{15., 5827.}	120.446	1.09883	newly 5 weeks cumul	3077.	3875.28	{48., 10550.}	140.106	0.887156	newly 68 weeks cumul	93253.	61048.3	{15133., 89885.}	34.5515	0.51429
18	newly 3 weeks spec	162.	423.98	{65., 917.}	192.358	0.385581	newly 3 weeks cumul	222.	776.02	{153., 1707.}	262.946	0.441441	newly 5 weeks spec	2807.	1365.36	{101., 3694.}	84.3285	0.793508	newly 5 weeks cumul	3654.	2938.62	{355., 7095.}	85.289	0.535998	newly 67 weeks cumul	99640.	71505.9	{46112., 92761.}	28.2357	0.159047
19	newly 3 weeks spec	726.	322.45	{203., 531.}	55.9711	0.385866	newly 3 weeks cumul	1060.	738.71	{490., 1235.}	38.7934	0.215147	newly 5 weeks spec	4054.	566.57	{356., 1042.}	86.0244	0.897856	newly 5 weeks cumul	6766.	1732.66	{1124., 2938.}	74.3917	0.62597	newly 62 weeks cumul	99318.	72903.8	{69449., 75932.}	26.5956	0.134904
20	newly 3 weeks spec	300.	926.32	{77., 1492.}	257.573	0.424248	newly 3 weeks cumul	402.	1493.25	{168., 2417.}	299.226	0.417549	newly 5 weeks spec	3692.	2461.72	{111., 5772.}	91.4919	0.769201	newly 5 weeks cumul	5182.	5688.46	{382., 12271.}	112.47	0.586148	newly 67 weeks cumul	98887.	74893.7	{50632., 92935.}	24.2633	0.131841
21	newly 3 weeks spec	120.	540.68	{10., 1249.}	396.767	0.67401	newly 3 weeks cumul	157.	895.08	{29., 1949.}	501.185	0.72776	newly 5 weeks spec	2417.	1911.1	{15., 5827.}	114.602	1.11783	newly 5 weeks cumul	3205.	3934.41	{48., 10550.}	136.975	0.893372	newly 68 weeks cumul	99034.	61346.7	{22643., 89885.}	38.0549	0.535477
22	newly 3 weeks spec	1137.	466.92	{165., 897.}	64.9657	0.514509	newly 3 weeks cumul	1829.	969.46	{394., 1784.}	56.2712	0.388677	newly 5 weeks spec	5958.	1047.53	{254., 2355.}	82.4245	0.930756	newly 5 weeks cumul	10630.	2726.61	{983., 5565.}	76.0501	0.727195	newly 64 weeks cumul	96878.	72163.1	{55530., 90260.}	25.5114	0.134401
23	newly 3 weeks spec	1235.	574.76	{168., 1214.}	65.5012	0.514363	newly 3 weeks cumul	1958.	1131.55	{416., 2150.}	57.9137	0.396805	newly 5 weeks spec	6598.	1486.66	{236., 3825.}	80.1761	0.881288	newly 5 weeks cumul	11490.	3568.02	{832., 8209.}	73.4348	0.692832	newly 65 weeks cumul	88617.	75641.6	{55883., 91727.}	16.8545	0.0856687
24	newly 3 weeks spec	102.	701.82	{77., 1486.}	600.51	0.548716	newly 3 weeks cumul	138.	1162.35	{168., 2331.}	746.312	0.661196	newly 5 weeks spec	2027.	2205.03	{118., 6464.}	115.839	0.650577	newly 5 weeks cumul	2665.	4745.38	{382., 12158.}	154.059	0.476973	newly 67 weeks cumul	98866.	74160.3	{48830., 92663.}	24.989	0.137826
25	newly 3 weeks spec	917.	384.8	{165., 591.}	60.2159	0.445812	newly 3 weeks cumul	1410.	855.71	{404., 1251.}	44.5284	0.284525	newly 5 weeks spec	3941.	743.74	{274., 1264.}	81.4783	0.818688	newly 5 weeks cumul	7263.	2138.14	{913., 3264.}	71.5911	0.604383	newly 63 weeks cumul	94839.	73263.9	{65364., 82662.}	22.7492	0.114229
26	newly 3 weeks spec	2138.	629.63	{311., 1028.}	70.5505	0.571791	newly 3 weeks cumul	3825.	1431.62	{753., 2226.}	62.572	0.46134	newly 5 weeks spec	6408.	1119.58	{501., 1950.}	82.5284	0.813402	newly 5 weeks cumul	14163.	3396.63	{1608., 5636.}	76.0176	0.663888	newly 61 weeks cumul	87857.	71544.3	{63354., 81230.}	18.5673	0.0911624
27	newly 3 weeks spec	1999.	462.49	{277., 802.}	76.8639	0.668266	newly 3 weeks cumul	3536.	1072.33	{638., 1761.}	69.6739	0.547794	newly 5 weeks spec	6511.	784.12	{452., 1470.}	87.957	0.960734	newly 5 weeks cumul	13890.	2461.14	{1448., 4348.}	82.2812	0.786194	newly 61 weeks cumul	98347.	71259.1	{62917., 75486.}	27.5432	0.141156
28	newly 3 weeks spec	310.	634.89	{22., 1455.}	193.661	0.682061	newly 3 weeks cumul	474.	1035.27	{35., 2439.}	184.428	0.588309	newly 5 weeks spec	2971.	2161.44	{24., 7494.}	112.354	1.11305	newly 5 weeks cumul	4384.	4512.28	{95., 15083.}	126.915	0.907488	newly 68 weeks cumul	89828.	63227.1	{24289., 90826.}	30.1493	0.388301
29	newly 3 weeks spec	609.	411.87	{131., 810.}	56.7373	0.354948	newly 3 weeks cumul	951.	827.34	{330., 1576.}	49.5394	0.2534	newly 5 weeks spec	3642.	1065.58	{232., 2789.}	75.101	0.749387	newly 5 weeks cumul	6162.	2566.51	{739., 5769.}	67.0514	0.552742	newly 65 weeks cumul	99052.	75081.2	{58436., 91713.}	24.2003	0.12749
30	newly 3 weeks spec	109.	529.33	{10., 1184.}	428.651	0.675644	newly 3 weeks cumul	138.	871.5	{29., 1848.}	560.884	0.75255	newly 5 weeks spec	1649.	1890.41	{15., 5827.}	141.401	1.0298	newly 5 weeks cumul	2255.	3875.28	{48., 10550.}	174.145	0.82418	newly 68 weeks cumul	99316.	61048.3	{15133., 89885.}	38.5313	0.541574
31	newly 3 weeks spec	3137.	1249.86	{1015., 1744.}	60.1575	0.408342	newly 3 weeks cumul	6051.	2913.33	{2393., 3989.}	51.8537	0.325099	newly 5 weeks spec	6713.	2038.28	{1605., 2843.}	69.6368	0.527442	newly 5 weeks cumul	17548.	6558.82	{5268., 9080.}	62.6235	0.435892	newly 58 weeks cumul	87424.	73473.6	{66077., 76573.}	15.9572	0.0765451
32	newly 3 weeks spec	1418.	439.69	{125., 865.}	70.1375	0.614964	newly 3 weeks cumul	2225.	929.77	{329., 1748.}	60.2324	0.465989	newly 5 weeks spec	5244.	982.64	{218., 2258.}	81.4851	0.893184	newly 5 weeks cumul	10289.	2577.24	{694., 5455.}	75.6276	0.724781	newly 64 weeks cumul	98867.	71758.3	{57414., 89900.}	27.4194	0.145512
33	newly 3 weeks spec	501.	534.05	{22., 1184.}	115.93	0.76265	newly 3 weeks cumul	673.	888.35	{35., 1949.}	115.208	0.613627	newly 5 weeks spec	5187.	1877.16	{24., 5827.}	91.9051	1.30464	newly 5 weeks cumul	7427.	3875.17	{95., 10550.}	94.4167	1.05458	newly 68 weeks cumul	99003.	60851.6	{15133., 89885.}	38.5356	0.492603
34	newly 3 weeks spec	1440.	400.25	{114., 816.}	74.184	0.679689	newly 3 weeks cumul	2363.	839.66	{301., 1561.}	67.113	0.546506	newly 5 weeks spec	6918.	898.15	{193., 2158.}	87.0172	1.07672	newly 5 weeks cumul	12627.	2345.11	{696., 5032.}	81.642	0.869626	newly 64 weeks cumul	95674.	72500.8	{56422., 90393.}	24.221	0.126537
35	newly 3 weeks spec	179.	540.68	{10., 1249.}	264.849	0.666688	newly 3 weeks cumul	229.	895.08	{29., 1949.}	329.31	0.652284	newly 5 weeks spec	2514.	1911.1	{15., 5827.}	112.495	1.12809	newly 5 weeks cumul	3425.	3934.41	{48., 10550.}	131.486	0.908222	newly 68 weeks cumul	95744.	61346.7	{22643., 89885.}	35.9263	0.520804
36	newly 3 weeks spec	451.	343.85	{105., 784.}	52.8004	0.311625	newly 3 weeks cumul	672.	725.03	{241., 1459.}	49.0789	0.213464	newly 5 weeks spec	3163.	768.4	{152., 1937.}	76.6759	0.792114	newly 5 weeks cumul	5077.	2013.33	{510., 4673.}	64.4347	0.543335	newly 64 weeks cumul	99501.	73826.	{62105., 89994.}	25.8038	0.13332
37	newly 3 weeks spec	142.	400.58	{117., 739.}	189.127	0.378828	newly 3 weeks cumul	189.	810.9	{269., 1521.}	329.503	0.548761	newly 5 weeks spec	2178.	991.31	{185., 1747.}	67.4559	0.557234	newly 5 weeks cumul	2952.	2433.55	{612., 4179.}	57.7327	0.324881	newly 65 weeks cumul	99424.	75019.8	{57154., 90986.}	24.5455	0.128754
38	newly 3 weeks spec	1570.	437.2	{238., 706.}	72.1529	0.596638	newly 3 weeks cumul	2591.	989.77	{563., 1539.}	61.7997	0.452153	newly 5 weeks spec	6266.	797.36	{396., 1443.}	87.2748	0.949441	newly 5 weeks cumul	12039.	2380.44	{1256., 3931.}	80.2273	0.746918	newly 62 weeks cumul	93543.	73418.3	{65634., 81942.}	21.5138	0.106327
39	newly 3 weeks spec	955.	452.94	{132., 836.}	58.9298	0.447098	newly 3 weeks cumul	1540.	935.09	{311., 1696.}	49.9682	0.33127	newly 5 weeks spec	4980.	1050.92	{200., 2284.}	79.3912	0.845764	newly 5 weeks cumul	8707.	2686.92	{649., 5268.}	71.3132	0.641577	newly 64 weeks cumul	97320.	75161.4	{60411., 90403.}	22.7688	0.116903
40	newly 3 weeks spec	1152.	554.64	{132., 1197.}	62.2483	0.497334	newly 3 weeks cumul	1821.	1087.12	{333., 2402.}	55.62	0.38913	newly 5 weeks spec	6088.	1428.49	{236., 3786.}	78.1414	0.857916	newly 5 weeks cumul	10814.	3423.11	{778., 7963.}	70.7308	0.678585	newly 65 weeks cumul	99384.	75636.2	{54368., 93451.}	23.895	0.125962
41	newly 3 weeks spec	84.	540.68	{10., 1249.}	578.881	0.718568	newly 3 weeks cumul	118.	895.08	{29., 1949.}	686.305	0.80111	newly 5 weeks spec	1333.	1911.1	{15., 5827.}	162.17	0.973909	newly 5 weeks cumul	1781.	3934.41	{48., 10550.}	210.702	0.779565	newly 68 weeks cumul	96330.	61346.7	{22643., 89885.}	36.3161	0.523454
42	newly 3 weeks spec	2385.	724.74	{568., 861.}	69.6126	0.523522	newly 3 weeks cumul	4744.	1702.12	{1363., 1983.}	64.1206	0.450453	newly 5 weeks spec	6151.	1213.09	{937., 1496.}	80.2782	0.712052	newly 5 weeks cumul	14873.	3862.01	{3060., 4618.}	74.0334	0.591418	newly 59 weeks cumul	96441.	73245.3	{69194., 76426.}	24.0517	0.120187
43	newly 3 weeks spec	942.	476.52	{127., 886.}	57.4607	0.435117	newly 3 weeks cumul	1522.	980.22	{309., 1786.}	50.5112	0.330226	newly 5 weeks spec	4281.	1110.31	{198., 2385.}	76.2231	0.77753	newly 5 weeks cumul	7799.	2822.25	{648., 5659.}	68.105	0.593147	newly 64 weeks cumul	98631.	75426.8	{59055., 90508.}	23.5263	0.121643
44	newly 3 weeks spec	717.	358.05	{111., 822.}	55.8647	0.421198	newly 3 weeks cumul	1097.	752.76	{274., 1579.}	48.1586	0.297359	newly 5 weeks spec	5449.	799.27	{177., 2218.}	85.3318	1.00019	newly 5 weeks cumul	8791.	2086.75	{601., 5093.}	76.2627	0.741912	newly 64 weeks cumul	99299.	73935.5	{60622., 90451.}	25.5426	0.132346
45	newly 3 weeks spec	514.	376.49	{85., 744.}	65.5973	0.421098	newly 3 weeks cumul	734.	726.59	{189., 1384.}	62.8488	0.308029	newly 5 weeks spec	4552.	1097.23	{142., 2520.}	83.1817	0.957506	newly 5 weeks cumul	6924.	2478.35	{452., 5183.}	75.6391	0.71262	newly 66 weeks cumul	99357.	72370.2	{47563., 92651.}	27.1615	0.151011
46	newly 3 weeks spec	247.	529.33	{10., 1184.}	193.47	0.688909	newly 3 weeks cumul	335.	871.5	{29., 1848.}	212.179	0.612518	newly 5 weeks spec	2717.	1890.41	{15., 5827.}	108.768	1.15562	newly 5 weeks cumul	3855.	3875.28	{48., 10550.}	122.267	0.937745	newly 68 weeks cumul	99095.	61048.3	{15133., 89885.}	38.3942	0.540607
47	newly 3 weeks spec	977.	393.07	{183., 582.}	61.6387	0.450918	newly 3 weeks cumul	1597.	869.49	{426., 1248.}	49.0025	0.315469	newly 5 weeks spec	4864.	762.27	{321., 1207.}	84.3283	0.87725	newly 5 weeks cumul	8755.	2184.92	{998., 3366.}	75.5308	0.659623	newly 63 weeks cumul	96045.	73553.1	{63390., 83316.}	23.4181	0.117904
48	newly 3 weeks spec	364.	415.33	{67., 763.}	77.1731	0.351862	newly 3 weeks cumul	527.	804.32	{180., 1427.}	89.4953	0.29898	newly 5 weeks spec	3183.	1219.88	{140., 2516.}	77.0393	0.774616	newly 5 weeks cumul	4879.	2748.83	{435., 5200.}	68.4368	0.546461	newly 66 weeks cumul	97052.	71167.	{43099., 92133.}	26.6713	0.150118
49	newly 3 weeks spec	1900.	522.29	{194., 981.}	72.8226	0.653226	newly 3 weeks cumul	3162.	1104.95	{509., 1914.}	65.6176	0.528151	newly 5 weeks spec	7611.	1170.46	{318., 2516.}	84.6215	0.965748	newly 5 weeks cumul	14764.	3062.26	{1044., 6028.}	79.2586	0.792667	newly 64 weeks cumul	95259.	71513.3	{54109., 90414.}	24.9275	0.132213
50	newly 3 weeks spec	943.	517.6	{146., 966.}	60.4136	0.428652	newly 3 weeks cumul	1533.	1026.07	{363., 1915.}	53.2935	0.330271	newly 5 weeks spec	5024.	1330.52	{252., 3045.}	76.0394	0.777956	newly 5 weeks cumul	8966.	3204.69	{841., 6379.}	69.5642	0.609011	newly 65 weeks cumul	98082.	76855.7	{58519., 91231.}	21.6414	0.112021
